# Spatial repellents: from discovery and development to evidence-based validation

**DOI:** 10.1186/1475-2875-11-164

**Published:** 2012-05-14

**Authors:** Nicole L Achee, Michael J Bangs, Robert Farlow, Gerry F Killeen, Steve Lindsay, James G Logan, Sarah J Moore, Mark Rowland, Kevin Sweeney, Steve J Torr, Laurence J Zwiebel, John P Grieco

**Affiliations:** 1 Department of Preventive Medicine & Biometrics, Uniformed Services University of the Health Sciences, 4301 Jones Bridge Road, Bethesda, MD 20814, USA; 2 Public Health & Malaria Control, Jl. Kertajasa, Kuala Kencana, Papua 99920, Indonesia; 3 R Farlow Consulting LLC, 156 Cardinal Cove Burkeville, TX 75932, USA; 4 Liverpool School of Tropical Medicine, Vector Group, Pembroke Place, Liverpool, L3 5QA, UK; 5 Department of Disease Control, Faculty of Infectious and Tropical Diseases, London School of Hygiene & Tropical Medicine, Keppel Street, London, WC1E 7HT, UK; 6 Office of Pesticide Programs, Registration Division, U.S. EPA, Washington, DC, USA; 7 Natural Resources Institute, University of Greenwich, Central Avenue, Chatham Maritime, Kent, ME4 4TB, UK; 8 Department of Biological Sciences, Vanderbilt University, Nashville, TN, 37232, USA

**Keywords:** Public health, Spatial repellents, Vector control, Vector behaviour modification

## Abstract

International public health workers are challenged by a burden of arthropod-borne disease that remains elevated despite best efforts in control programmes. With this challenge comes the opportunity to develop novel vector control paradigms to guide product development and programme implementation. The role of vector behaviour modification in disease control was first highlighted several decades ago but has received limited attention within the public health community. This paper presents current evidence highlighting the value of sub-lethal agents, specifically spatial repellents, and their use in global health, and identifies the primary challenges towards establishing a clearly defined and recommended role for spatial repellent products in disease control.

## Background

**Figure 1:**
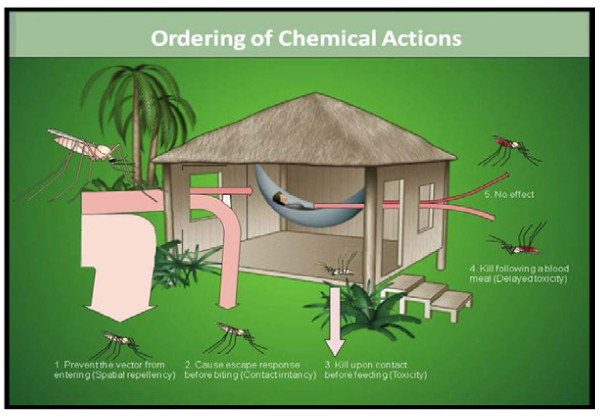
**The general concept of spatial repellency is clear:** to prevent an arthropod from entering a space occupied by a potential human host to reduce encounters between humans and vectors thereby eliminating or reducing the probability (risk) of pathogen transmission to either insect or human.

Arthropod-borne diseases, such as malaria and dengue, remain significant health problems worldwide despite decades of organized vector control [1,2]. The reasons for this are complex and include both limited option and availability of active ingredients (AIs), and a lack of understanding of all actions and mechanisms that such AIs exert against the target insects (Figure 1). A better understanding of such actions would help in the design of alternative application formats for global vector control strategies beyond the current choices of insecticide-treated bed nets (ITNs) and indoor residual spraying (IRS). For decades, research and development in vector control have taken a secondary position to development of other methods of disease control – namely chemotherapy and vaccines. Simultaneously, the focus of efforts in vector control was on ITNs with minimal emphasis on other vector control strategies. As a consequence, there is now an urgent need to improve current tools and advance the development of novel products based on new paradigms that function through alternative mechanisms of action – i.e., vector behaviour modification that specifically includes spatial repellency (SR).

Currently, there are 15 AI compounds recommended by WHO for adult insect vector control restricted to only four chemical classes, with the most recent addition - etofenprox (pyrethroid) - occurring in 1999 [3,4]. Twenty years on, the global community continues to place expectations of population-level protection using these very same, limited groups of actives in the same way on walls of houses and bed nets. Although this limited arsenal of AIs and application modalities has contributed to decreasing the malaria burden, it is becoming grossly inadequate to sustain reductions in disease burden in many endemic countries. This is due, in part, to an overreliance on pyrethroids (which dominate the WHO shortlist of approved AIs) in both public health and agriculture and a resultant increasing occurrence of insecticide resistance, coupled with variable and poorly understood ecologies of different vector species [5].

No new classes of traditional vector control insecticides have been developed in recent decades (excluding reformulated active ingredients), therefore there is reason to assume that very few such compounds, if any, are currently in the development pipeline and expected for use in the near future. New AIs that have been explored and are ripe for development lack the important characteristic of repellency [6,7]. It is these significant behavioural effects, and alternative mechanisms of action that should be exploited for the development of innovative vector control products to better manage current and mitigate future insecticide resistance problems. The wider effort could be enhanced by broadening the scope of AI discovery to include screening criteria that identify compounds and/or chemical classes that exploit behavioural modification as a means to disease reduction [8].

The use of spatial repellents to create a vector-free space, thereby preventing contact between human and vector, thus preventing disease transmission, is demonstrably effective [9-22]. Yet, use of spatial repellency is neither endorsed nor recognized as a component of a multilateral disease control strategy. There are multiple rationales that argue in favour of a change in this policy. Benefits of sub-lethal over more conventional lethality-directed chemical approaches include: 1) marketable for insecticide-management purposes because its useful for delaying the onset of resistance to active ingredients used for ITNs or IRS; 2) effective for outdoor protection, something that IRS and ITNs have little impact on; 3) useful in attacking other components of vector behaviour such as pre, during and post-host-seeking, i.e. to disrupt critical behavioural sequences that can prevent blood-feeding (and disease transmission) and strengthen the effectiveness of integrated vector control strategies; 4) employable against multiple vectors, behaviours and species – not just those that feed and rest inside houses -and subsequently against other arthropod-borne diseases, and 5) useful against economically important insects, especially agricultural pests, where market forces will fuel the cost of AI discovery and development. Indeed, strategies such as a push-pull system that integrates repellents or mating disruptors with attractants and trapping methods, have successfully been implemented for agricultural pest control and are currently under investigation for vector control [23].

Incentives for changing the prevailing screening and evaluation paradigms for chemical control products should be driven through an evidence-based approach. Over the past several years, four formal national and international meetings [24] were convened to bring together academics, industry and global public health experts, including representatives from the WHO and the WHO Pesticide Evaluation Scheme (WHOPES) [25], to discuss the role of spatial repellent chemicals, whose effects are not reliant on acute toxicity or lethality, in the reduction of arthropod-borne diseases. A critical aspect of these meetings and subsequent efforts has been to establish a critical path of development for these products (SRCPD). The principal goal of the SRCPD is to gain formal acceptance of the requirement for the development and incorporation of spatial repellent-based strategies as integral components for disease vector control from global health authorities such as WHOPES. As such, the adoption of a widely accepted SRCPD is expected to create opportunities and impetus for industry, academia and other private/public sector entities to increase ongoing efforts to discover, validate and develop novel repellent AIs that represent classes of chemicals that focus on vector behavioural modification rather than toxicity/lethality as well as find new means of utilizing existing compounds in behavioural disruption. This strategic document is expected to aid in a comprehensive effort to develop and eventually deploy innovative control methods for either stand-alone products and/or integrated interventions for combating vector-borne diseases.

The intention of this paper is to disseminate key outcomes of the core working group, to highlight known and potential benefits of spatial repellency, identify specific obstacles and challenges to the successful development of spatial repellent tools, and to highlight key components of the SRCPD needed to achieve the goal of recommending spatial repellents as a viable means for disease prevention (Table 1).

**Table 1:** Summary points outlining role of spatial repellents and requirements for adoption in vector control

**Summary points**	
· The discovery, development and use of novel vector control tools will be required to achieve the goal of malaria elimination and eradication	
· Evidence exists of the benefits of sub-lethal approaches for interrupting human-vector contact but epidemiological data is insufficient to influence policy-makers to recommend spatial repellent tools for disease control confidently	
· The adoption of a new paradigm shift in vector control to include behavior modification will require a new set of laboratory and field assay tools, standardized endpoints and analyses which must also be endorsed and adopted by leading global public health authorities	

### Making choices: repel or kill? Evidence of value

**Figure 2:**
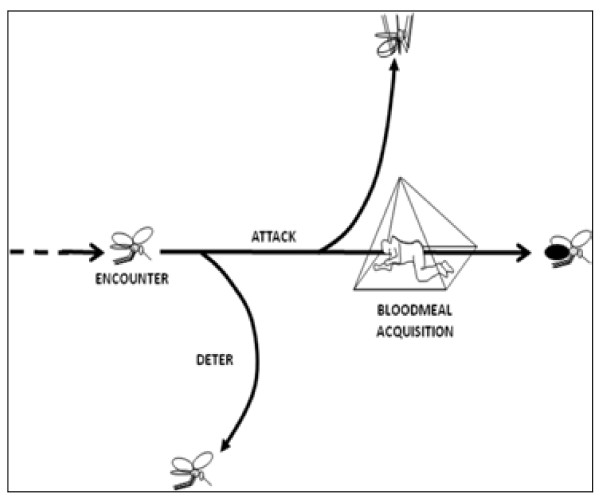
**The fundamental choice between killing mosquitoes and deterring them:** mosquitoes that abort attacks on humans because of sub-toxic exposure are, by definition, not exposed to toxic levels that kill them (Killeen GF and Moore SJ with permission).

Spatial, or area repellents (also known as deterrents [26]) are defined here as chemicals that work in the vapor phase to prevent human-vector contact by disrupting normal behavioural patterns within a designated area or “safe zone” (e.g. a space occupied by potential human hosts) thus making the space unsuitable for the insect (Figure 2). Depending on efficacy of the AI and application modality, this would result in a vector-free (or greatly reduced / suppressed) area. The unique benefit of SR is that the safe-zone can include specific areas both indoors and outside. The volume of space that is ‘protected’, or minimum protection range, will be dependent on the properties of the AI, application platform and/or environmental conditions (e.g. air flow, temperature and humidity). Regardless of the particulars, the general concept of spatial repellency is clear: to discourage an arthropod from entering a space occupied by a potential human host thus reducing encounters between humans and vectors thereby eliminating or reducing the probability (risk) of pathogen transmission to either insect or human.

The current prevailing strategic paradigm in vector control is that effective delivery of an acutely toxic compound will have the greatest impact for disease suppression by reducing the survival of the overall vector population, a concept based on the classic Ross-Macdonald model used for advocating DDT IRS during the Global Malaria Elimination Programme in the 1950s-1960s. If correct, what is the rationale for removing or otherwise deterring a vector from a specified space without directly killing it? If spatial repellency to prevent bites were to be the primary control mechanism, malaria transmission will be reduced if mosquitoes 1) are diverted to alternative non-human hosts which cannot carry malaria, and/or 2) feed, reproduce and survive less because humans are difficult to access and no alternative source of blood is available [19]. The result will be reductions in numbers of both humans and vectors being infected and this result has a dramatic effect on mathematical models of malaria transmission [27].

The immediate advantages of modifying vector behaviour that results in movement away from a human host, is a delayed or diminished development in the emergence of insecticide resistance by minimizing the intensity of selection pressure from contact-mediated toxicity mechanisms as well as the potential reduction of toxic effects of a chemical to human and non-target organisms. The added long-term benefits of demonstrating disease impact of spatial repellents include the discovery and development of new chemical active ingredients and/or new modes of action that target and exploit the normal vector behavioural patterns outside and surrounding the home while in search of a host. A better understanding of vector behaviour in this context can stimulate innovative product development and enhance vector control. The accumulated long-term effects of such deterrent events upon mosquito life histories can reduce malaria transmission by forcing mosquitoes to either feed upon non-human hosts or to search more broadly for alternative blood (and subsequently oviposition sites), thereby reducing human blood indices, vector survival, feeding frequency and reproduction rates. It is likely that, the longer a vector remains exposed to harsher and more demanding outdoor conditions the more likely it is that the vector will die. Outdoors, vectors risk greater predation, physiological stressful environments, and excessive energy expenditure during host-seeking, or identifying a resting or oviposition site [19]. In essence, the vector population will potentially experience greater adverse environmental exposure and therefore mortality without chemically induced selection pressure thereby potentially increasing the sustainability of existing and novel chemical interventions. Furthermore, vector populations that survive exposure to sub-lethal, spatial repellents may subsequently show permanent or semi-permanent disruption of host-seeking and blood-feeding behaviours [28]. The reduction in host-contact/feeding success could ultimately lead to reduced overall numbers and survival of older mosquitoes that transmit mature infectious stage parasites, thereby suppressing transmission at the community level –a more subtle means to achieve the desired outcome of traditional adulticidal strategies [19].

The case for developing a SRCPD is strengthened by several research programmes that have, and continue, to generate evidence of the benefits of sub-lethal approaches for disease control. Studies evaluating physical barriers (e.g. house screening and untreated bed nets) have shown reduction on disease burden in the absence of vector lethality [20,21]. Specifically, a randomized controlled trial where screening houses resulted in a 50% reduction in malaria vectors entering the house, produced a 50% reduction in anaemia in young children. In this study, houses were screened with untreated netting and, since they ‘repelled’ mosquitoes and did not kill them, they were protected in a similar manner to that expected for spatial repellents. Further evidence exists describing the potential effects of sub-lethal toxic chemicals and disease reduction [9-22]. Indeed, beginning in the 1940s, numerous observations were made on the ability of DDT (arguably the most effective chemical tool so far developed to reduce arthropod-borne disease burden in history) to create a vector-free space [29]. When DDT was sprayed on the interior wall surfaces of houses, there were essentially no mosquitoes to be found indoors, with malaria rates subsequently declining dramatically and vector populations were reduced overall [10,11,17]. Those results are attributed primarily to the spatial repellent action of DDT (a significant and generally underrated property) and not the toxic action alone [30]. This conclusion is based on numerous observational and quantitative studies that clearly indicate the primary action of DDT is spatial repellency with ‘irritancy’ (contact excitation) and toxicity as secondary and tertiary effects of lower order [12-19]. Furthermore, a dramatic and unmistakable reduction in disease incidence has been documented following IRS with DDT even in areas with significant DDT-resistant vector populations [31]. This paper is not designed to argue the use of DDT in vector control programs. Instead, it aims to highlight the spatial repellent characteristics of the AI to provide a significant example of the role and value of behavior modification in disease control.

A paradigm shift from contact toxicity-based strategies to a broader approach using behaviour-modifying AIs and modalities that can operate safely at a distance, will require clear evidence that demonstrate: 1) chemicals can exert behaviour-modifying characteristics relating to vector/host interactions (via entomological validation); 2) that peripheral exposure to such chemicals is not harmful or otherwise unfavourable to humans, and 3) sub-lethal approaches to vector control can significantly impact disease transmission (via epidemiological validation). Of these, chemical-based behaviour modification has been comprehensively demonstrated under both laboratory and field conditions [12-16]. Although historical data exists supporting the associated sub-lethal effects on disease risk reduction [10,17], no controlled study design has been implemented to specifically correlate spatial repellency actions with direct, real-time impact on disease incidence.

## Expectations

Human populations have long been aware that using personal repellents and deterrents can reduce biting from blood-sucking insects and utilize these materials widely, even when associated with marked financial costs [32]. In economies that can support even minimal discretionary spending, it is not uncommon to observe use of electric fans or topical repellents to reduce biting burden and/or the purchase of various commercial products, including insecticidal powders, aerosols, nets, and coils that function by toxic (acute-kill) chemical actions. Spatial repellent actives could be integrated into such consumer products or used to enhance IRS and ITN programmes, where appropriate, to provide added protection to individuals, households and communities using AIs with minimal mammalian toxicity. However, the public health community must think beyond current formats and consider novel consumer-based products that increase market value and thereby compliance and sustainability by combining end-user “wants” (i.e. products that provide utility or beautification, such as decorative mats, clothing, etc.) with vector control thereby creating opportunities for innovative, cost-effective and affordable vector control delivery platforms. Marketing these tools through a consumer product channel poses a viable solution to managing burden of product delivery to target populations [32].

**Figure 3:**
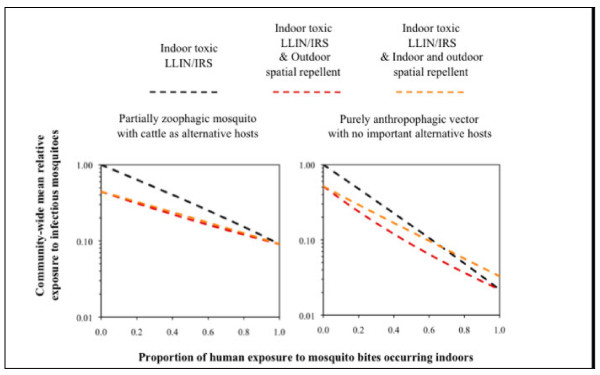
**An outdoor role for spatial repellents?** Mathematical models show that the best strategy for application of spatial repellents depends on the vector. Indoors and outdoors use is best for those vectors least susceptible to LLINs/IRS, while use of spatial repellents outdoors is best to complement LLINs/IRS in areas where vectors feed indoors on humans (Killeen, GF and Moore, SJ with permission).

It is realistic to conceptualize a spatial repellent product that can be adapted to exterior areas of homes or within the immediate peri-domestic environment to effectively protect a wider spatial area from pathogen transmission throughout both evening and daytime hours. In fact, in the face of elimination/eradication of malaria, it is becoming more evident that those vectors with behaviours which are not controlled by conventional IRS and ITNs or spaces where physical structures are absent, will become the focus of residual transmission and will be the barrier to success or failure [26,33]. When one considers the human population at risk of transmission outdoors, the niche for spatial repellents becomes increasingly evident due to the lack of current control products for those humans becoming infected in these areas [34,35] (Figure 3). What tools we currently have are not enough. The role of combined organized vector control (IRS and ITNs) with personal protection (consumer products) to enhance human protection from infection is upon us and where SR could equally be useful.

Such products could be disseminated in a variety of delivery platforms: as stand-alone consumer products or designed to be integrated into community-based or vertically organized vector control programmes. By applying separate products with very different, complementary modes of action to distinct fractions of transmission sites, it may be possible to both extend rather than duplicate coverage of transmission *per se* and exploit the benefits of SR against direct-toxicity. Given that repellent actions and mechanisms are independent of toxicity and the necessity for direct contact with a treated surface is averted, the authors envision a spatial repellent active ingredient that is effective against various genera and species of disease vectors, either insecticide resistant or susceptible, that can be adapted to a plethora of conditions.

### Hurdles to overcome

In order for a spatial repellent product to enter into the market, a set of development criteria must be met. These include measures related to scientific, regulatory and social parameters. In part, these criteria will comprise the SRCPD and outline the endpoints of a target product profile for a spatial repellent product. The authors have chosen to describe three major hurdles identified in the SRCPD (Table 2).

**Table 2:** Key components of a spatial repellent critical path of development (SRCPD)

**Key components of a SRCPD**	
1 Proof-of-Principle: demonstrating a spatial repellent will impact disease at the community level	
2 Correlating entomological endpoints with reduction in infection incidence rates using repellent tools	
3 Measuring the impact of diversion of repelled vectors to untreated sources under varying transmission dynamics	
4 Defining the limitations of spatial repellency in both susceptible and insecticide resistant vector populations	
5 Developing standardized protocols and measures for the evaluation of vector behavior modification as it relates to host-feeding following exposure to spatial repellents (i.e., host-seeking, feeding, resting, and oviposition) to identify long-term effects of spatial repellents	
6 Engagement and recruitment of industry and academic partners to adopt standardized protocols and measures for the screening of chemical AIs to include spatial repellency	
7 Identifying the underlying genetic/neurobiological basis of vector behaviors to provide insight into the rationale design of new repellents	

1. **Generation of sufficient epidemiological data to influence policy-makers to recommend the incorporation of spatial repellents into current multi-lateral disease control programmes confidently.** The concept of spatial repellency will be accepted once indisputable proof-of-principle that a spatial repellent can reduce human disease through sub-lethal chemical actions is provided. While associations between SR and reduced disease transmission have been made, they generally exist in two incomplete formats: epidemiological data post-chemical treatment that lacks a sufficient entomological component or evaluation of changes in entomological endpoints due to repellency without supporting case data that measures disease impact [9-22]. To date, no published accounts linking epidemiological and entomological components exist for spatial repellents, thus there is a critical need for Phase III community trials integrating simultaneous monitoring of infection incidence with vector population metrics (i.e. parity, sporozoite rates, blood meal indices, abundance etc.), one of which is currently underway. Such confirmatory studies will require unambiguous entomological measures of repellency *versus* irritancy and/or knock down effects in reducing vector entry into a given interior space or outside area, as well as reductions in vector biting densities (to include potential redirection to untreated spaces with human hosts) concurrent with reduced pathogen transmission. The challenge arises when designing an impact study to ensure both entomological and parasitological endpoints correlate with true repellency effects.

2. **Identification and validation of the entomological end points that predict a public health impact using spatial repellency.** There are three AIs currently registered by the USEPA for outdoor use as vaporizing spatial repellents (allethrin, pyrethrin and metofluthrin). Prallethrin, permethrin and cyflutrhin when used as aerosols and/or surface sprays also have SR claims. However, the endpoint used to label them as spatial repellents is anti-biting. These pyrethrum and pyrethroid-based products are all known to have knock down and toxicity at defined concentrations. These products may be sufficient to achieve our public health goals. If they are not, then effective screening systems will have to be established. A critical element of that process will be establishing end points. Currently, there is no consensus about what those end points may be and the best means to quantify them. Critical endpoints may include reduced entry into a treated space, reduced abundance within a space, and enhanced exit from a space. Furthermore, the end points will have to be accurately quantified. How far must any of those measures have to shift in order to create a reduction in pathogen transmission? How do shorter 24-hour measures of those parameters, currently used in routine testing, relate to the cumulative impact over the days or weeks of a transmission season? Once the end points for evaluating SR have been established (identified and validated) effective screening can begin. We will need to lobby our industry partners to engage in the systematic screening of spatial repellency in either existing chemical libraries and/or discovery of novel chemical classes for vector control.

3. **Motivating an objective, universally acceptable paradigmatic shift in the current screening protocols for the assessment and discovery of chemical vector control products.** In order to generate spatial repellent products, the scientific community must have well defined, effective active ingredients. Current laboratory screening protocols utilize knock down and mortality as sole criteria for advancing compounds to the next level in the screening process [8]. Even with disease impact studies and a vast market potential for repellent products there will be a requirement to adopt, via scientific community consensus, a new set of laboratory and field assay tools, standardized endpoints and analyses. These must come from a consensus within the scientific and development communities and must be sufficiently clear and uniform to provide reliable and reproducible results to evaluate behavioural responses of a candidate spatial repellent. In order to promote worldwide recognition of spatial repellents for use in vector control, these new testing protocols and overall evaluation schemes must also be endorsed and adopted by leading public health authorities such as WHOPES. Currently, WHOPES has standard evaluation protocols for insecticides as contact irritants and toxicants; however, there is an absence of standard protocols for evaluating the behavioural effects of spatial repellent AIs with corresponding endpoints. It is expected that a screening cascade will need to be developed to allow the step-wise identification of candidate compounds. This cascade should exploit each assay’s sensitivity in an ordered, procedural manner. For example, the process could begin with high-throughput to include an intracellular evaluation (i.e., gene expression and/or electrophysiological responses) or novel molecular target that can be screened via heterologous expression platforms. Discovery-based efforts would then advance to a laboratory and then semi-field behavioural systems, through to controlled experimental hut studies under field conditions. One essential part of this process will be to define the mode(s) of action of active ingredients that elicit the desired behavioural effect. This fundamental information will aid the development of effective and efficient screening tools and means of exploiting new AIs. The promising news is that there are several electrophysiological and behavioural assays with correspondingly robust analyses currently being used in vector biology research that could be incorporated into such schemes once adopted by WHOPES and the wider scientific community [16,36-42].

### Future directions

The ultimate goal of this working group is to provide a basis for the recommendation by public health authorities for the incorporation of spatial repellent products in multi-lateral efforts focused on disease vector control. The success of this effort will depend on a concerted effort between public health entities, regulators, industries, non-governmental agencies and sponsors, and academic partnerships coordinated through a formal consortium which the authors now propose: Advancing Repellency to Recommendation Consortium (ARRC) to facilitate the planning, implementation and data dissemination for priority research studies as outlined in the SRCPD. The focus of ARRC will be to continue developing a structured effort to increase the number of potential and efficacious spatial repellent tools following a precise SRCPD format beginning with the identification of priority research areas. Combined, datasets from these studies will provide insight into the rational design of new repellent active ingredients, establish critical baseline data and generate consensus on essential outcome measures and data interpretation required for evaluation of the efficacy of repellent products and control strategies.

## Summary

Despite many decades of concerted effort along a number of fronts, the long and difficult battle to control vector-borne diseases continues. It is incumbent on the international disease control community to step up to this challenge and embrace both the need and opportunity for innovation. It is accepted this includes not only the attempt to use available tools optimally but also develop new ones to improve vector control. The authors take the view that control of pathogen transmission by preventing vectors from entering human-occupied spaces is both beneficial and cost-effective and that efforts to dramatically increase investments and efforts to develop novel spatial repellent tools and strategies are urgently needed.

Paradoxically, the value of vector behaviour modification in disease control has been recognized for many decades but largely under-appreciated. As a consequence, it is likely that potentially effective chemicals and novel products have been missed or overlooked. With current efforts focused on the elimination and eventual eradication of vector-borne diseases such as malaria, this must change [43]. It is the authors’ goal that this article will bring renewed awareness, stimulation and focus to the importance of spatial repellency as an effective tool in the fight against vector-borne disease transmission. By doing so it is hoped that further discussion and a sustained investment in R&D will be quickly forthcoming in order to bring a new generation of effective chemicals and novel products into the disease control/eradication armamentarium.

## Competing interests

RF serves as an independent consultant for BASF The Chemical Company, involved in vector control product R&D. NLA, JPG and RF are currently applying for patents through BASF relating to the content of this manuscript. The following authors have received funding support for research projects from manufacturers of insecticidal public health products: Vestergaard Frandsen SA (GFK), Syngenta (SJM), Pinnacle Development (SJM) and SC Johnson (SJM, NLA and JPG). Some research by JGL is funded by industry via the London School of Hygiene and Tropical Medicine Arthropod Control Product Test Centre (arctec) which offers consultancy and the evaluation of control technologies, including repellents. JGL is also a co-inventor on a patented insect repellent technology. LZ holds several patents and patent applications concerning the composition and use of novel chemicals discovered for insect control via a Bill & Melinda Gates Grand Challenges-funded project. LZ is currently in discussions with the private sector for developing these leads into products. MJB, SL, MR, KS, SJT declare that they have no competing financial interests.

## Authors’ contributions

NLA drafted the original version of the manuscript. MJB, RF, GFK, SL, JGL, SJM, MR, KS, ST, LZ and JPG participated in the revision of the manuscript. All authors read and approved the final manuscript.
